# Iodine and Its Impact on the Immune System of Humans and Domesticated Mammals: A Narrative Review

**DOI:** 10.3390/nu18152432

**Published:** 2026-07-25

**Authors:** Rachael A. Simpson, Umesh K. Shandilya, Lauri C. Wagter-Lesperance, Byram W. Bridle, Bonnie A. Mallard, Niel A. Karrow

**Affiliations:** 1 Department of Animal Biosciences, University of Guelph, Guelph, ON N1G 2W1, Canada; rsimps11@uoguelph.ca (R.A.S.); ushand@uoguelph.ca (U.K.S.); 2 Department of Pathobiology, University of Guelph, Guelph, ON N1G 2W1, Canada; lwagterl@uoguelph.ca (L.C.W.-L.); bbridle@uoguelph.ca (B.W.B.); bmallard@uoguelph.ca (B.A.M.); 3 ImmunoCeutica Inc., RPO Clair, P.O. Box 27069, Guelph, ON N1L 0C1, Canada

**Keywords:** autoimmune thyroid disease, dose-dependent, iodine, iodide, leukocytes, thyroid hormone

## Abstract

Iodine is an essential micronutrient required for coordinating thyroid hormone synthesis, growth, neurodevelopment, metabolism, and immune function across humans and domesticated mammals. Molecular iodine can act as an antioxidant, anti-inflammatory, and anti-proliferative agent in several tissues, but these mechanistic effects require confirmation in well-designed human studies. Recommended intakes fall within a narrow optimal range, reflecting a U-shaped response curve in which adequate intake supports systemic homeostasis, while both deficient and excessive intakes can impact immune function. Chronic deficiency impairs neurodevelopment and reproductive performance and weakens the host microbial defences, while sustained excess promotes oxidative stress, alters cytokine profiles, and in genetically susceptible individuals, increases the risk of subclinical or overt thyroid dysfunction and autoimmune thyroid disease. This review summarizes iodine nutrikinetics, dose-dependent outcomes and susceptibility in humans and domesticated mammalian species, emphasizing the importance of maintaining appropriate iodine status to support immunocompetence while minimizing adverse effects.

## 1. Introduction

Iodine is an essential micronutrient required for several physiological processes across vertebrate species. Iodine is concentrated by the thyroid gland, but also accumulates in the salivary glands, gastric mucosa, lactating mammary gland, reproductive organs, and leukocytes [1]. Iodine influences thyroid hormone (TH) production, mucosal defence, and leukocyte functions [2]. Vertebrates are mainly exposed to iodine through dietary intake, including inorganic iodide (I^−^) and iodate (${IO}_{3}^{-}$), as well as smaller amounts via antiseptics, contrast media, or supplements in the form of potassium iodide, Lugol’s iodine or povidone-iodine [3]. In humans, iodine is obtained primarily from fish, seaweed, dairy products, eggs, iodized table salt, and dietary supplements, whereas, in domesticated mammals, it is supplied through fortified feeds, iodized salt licks, and commercial feeds [4].

Adequate levels of iodine are required during several critical life stages, including early developmental periods of rapid growth and development, pregnancy, and in individuals with pre-existing thyroid disorders [1]. Across humans and domesticated mammals, iodine status has a narrow optimal range; as such, deficient and excessive intakes can have profound effects on endocrine and immune system homeostasis [1]. Chronic iodine deficiency significantly reduces TH production, leading to impaired neurodevelopment, reduced growth and reproductive performance, and increased susceptibility to microbial infections [2]. In contrast, excessive iodine exposure promotes oxidative stress, alters inflammatory cytokine profiles, and has been associated with genetic and epigenetic variants that promote autoimmune thyroid diseases in genetically susceptible individuals [1,5]. Thus, adequate intake of iodine supports optimal endocrine and immune functions [1].

Even where global or national iodine guidelines exist, intake recommendations and age or life stage categories, particularly for children (0–13 years) and pregnant or lactating women, are defined differently across documents, and do not always specify the same values; the human values summarized in Table 1 follow the Canadian Dietary Reference Intakes tables and the Institute of Medicine (IOM) Panel on Micronutrients, which most closely reflect current North American practice [6,7]. The World Health Organization (WHO) and the American Thyroid Association (ATA) also detail the criteria and evaluation of dietary, physiological, and epidemiological factors used to define their iodine intake recommendations and life stage categories [8,9]. Differences in daily iodine requirements and upper limits are summarized by species and stage of development in Table 1.

Nutritionally relevant concentrations of iodine are traditionally defined using population-level relationships between intake, biomarkers such as urinary iodine or TH levels, and the prevention of overt deficiency disorders, leading to recommended ranges that are largely anchored in thyroid and neurodevelopmental outcomes for broad life stage categories [6,7,9,10,11,12,13,14,15,16,17,18,19]. Across species, current iodine guidelines are incomplete and oversimplified, as formal recommendations either do not exist for some species and physiological states, or a single value is applied across life stages regardless of changes in endocrine, developmental, or immunological demands [10,11,12,13,14,15,16,17]. Where guidelines do exist, they are typically derived from thyroid activity, such as TH concentrations, goitre prevalence, and growth performance, and rarely consider extrathyroidal targets of iodine accumulation, or how these tissues respond to deficiency, or sustained iodine excess [20].

Emerging evidence indicates that iodine, including molecular iodine, may induce epigenetic changes and exert context-dependent immunomodulatory effects at doses within or near these “adequate” nutritional ranges [21,22]. This suggests that the window compatible with optimal immune and epigenetic regulation may be narrow and not fully captured by current dietary reference values; however, mechanisms still need to be verified in human studies [21,22]. Consequently, while iodine homeostasis must be maintained within a relatively tight interval to avoid both deficiency or excess, the precise ranges of this interval for specific immune endpoints remain incompletely defined and may differ from the ranges established to solely prevent classical thyroid dysfunction [23,24]. As a result, the dose-dependent effects of different iodine compounds, optimal concentrations and exposure levels remain poorly characterized and inconsistently integrated across species [25]. The lack of evidence-based recommendations represents a key knowledge gap that limits vulnerable individuals and animals with pre-existing thyroid conditions [1,26]. The studies discussed throughout this manuscript span in vitro models, animal experiments, and clinical or epidemiological data, and, therefore, require careful consideration of how experimental iodine exposures relate to tissue bioavailability and immune responses in vivo. This review outlines how iodine-based compounds directly and indirectly modulate the immune function of humans and domesticated mammals, with a focus on dose-dependent mechanisms of immunomodulation and outcomes of deficiency and toxicity across species. Domesticated mammals were included to evaluate and clarify which iodine-related immune effects can be considered general across mammals, and which are more likely to be species-specific; this approach also offers a One Health perspective to the review.

## 2. Literature Search and Selection Strategy

The relevant literature on iodine exposure, thyroid function, and immune or oxidative outcomes was identified through targeted searches in PubMed, supplemented by manual screening of reference lists from key reviews and guidelines. Searches combined terms for iodine and iodinated compounds (iodine, iodide, molecular iodine, povidone-iodine, iodine excess, iodine deficiency) with endocrine and immune concepts (thyroiditis, autoimmune thyroid disease, hypothyroidism, innate immunity, adaptive immunity, oxidative stress), limited to articles with accessible full text, with a preference to articles published from 2020 or later, plus earlier foundational studies that outline key mechanisms that are consistent with more recent studies.

Records were included when they provided primary data or a clear synthesis relating iodine status, excess, or supplementation to thyroid or immune outcomes, or when they specified iodine intake recommendations or maximum allowable levels in humans or domestic animals. Editorials and opinion pieces without data or explicit synthesis, and articles focused solely on non-iodine trace minerals, were excluded. Human studies were used to define exposure ranges, inflammatory and mechanistic pathways; animal studies (cattle, sheep, goats, rabbits, dogs, cats, experimental rodents) were included where they examined iodine effects on thyroid or immune function. Regulatory and nutritional guideline documents provided species-specific context for “adequate” versus “excessive” iodine exposure.

In this manuscript, we use the term iodine in a general sense to refer to iodine exposure or iodine status, irrespective of chemical form, unless otherwise specified. Iodide (I^−^) refers to the reduced, inorganic anion that is the predominant circulating form absorbed from diet and used by the thyroid [23,24]. Molecular iodine (I_2_) refers to the non-ionic form used in antiseptics and some supplements, including the preparations discussed throughout this manuscript [25]. Where mechanistic or molecular effects depend on the specific chemical form, we have named the relevant species and avoided using the generic term “iodine”, alone. Throughout, “iodine status”, or “iodine intake”, are used for population and nutritional data that do not distinguish chemical species, whereas experimental sections specify the exact form and concentration administered [6,7].

## 3. Nutrikinetics of Iodine and TH Transport

**Figure 1:**
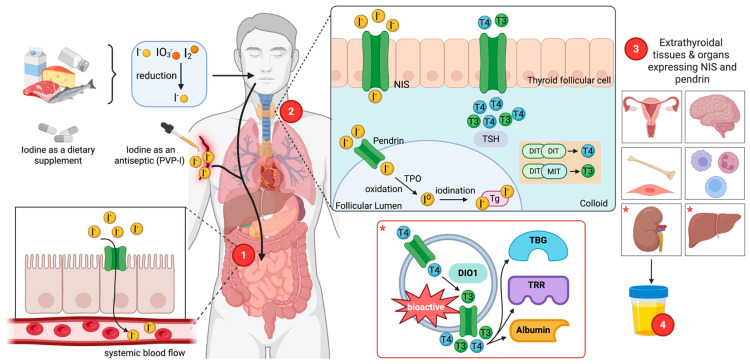
Iodine nutrikinetics and the thyroid hormone pathway. Dietary and supplemental iodine, as iodide (I^−^) or iodate (IO_3_^−^ reduced to I^−^), is absorbed in the small intestine and enters the systemic circulation, where iodide is taken up by sodium iodide symporter (NIS)-expressing tissues. In thyroid follicular cells, basolateral NIS and apical pendrin mediate I^−^ transport to the follicular lumen, where thyroid peroxidase (TPO) catalyzes oxidation and iodination of thyroglobulin (Tg) tyrosine residues to form monoiodotyrosine (MIT) and diiodotyrosine (DIT), followed by coupling to generate thyroxine (tetraiodothyronine, T4) and triiodotyrosine (T3) that are secreted into blood under thyroid-stimulating hormone (TSH) stimulation. Circulating thyroid hormones bind to carrier proteins, while in peripheral tissues such as liver and kidney, type 1 iodothyronine deiodinase (DIO1) converts T4 to bioactive T3, supporting hormone-dependent actions in target organs including the brain, bone, reproductive tissues, leukocytes, and muscle. Excess iodide is cleared predominantly by the kidney via urinary excretion. Asterisk (*) indicates that the T3 activation occurs in the liver and kidneys.

Iodine is ingested as molecular iodine, iodide, and iodate from drinking water, iodized salt and iodine-enriched supplements by humans and domesticated mammals [27]. Iodine and iodate are reduced in the stomach and duodenum to iodide, which is the only form that can efficiently cross specialized transporters such as sodium iodide symporters (NISs) [28] (Figure 1). Dietary iodide is absorbed mainly in the small intestine; recent studies indicate that NISs on the apical membrane of enterocytes mediate active iodide uptake from lumen to blood [2]. However, several medical conditions have contaminants that can suppress NIS expression, and dietary restrictions can affect how efficiently iodine is absorbed [1,7]. Once in circulation, iodide is transported throughout the body and absorbed by organs and tissues that express either NISs, or pendrin, a transmembrane anion exchanger protein [2,29].

The thyroid contains up to 80% of the iodide required for TH synthesis (Figure 1) [29]. Thyroid follicular cells express NISs on the basolateral membrane to transport iodide from the circulation into the thyroid, where thyroid peroxidase (TPO) oxidizes iodide and binds it to the tyrosyl residues on thyroglobulin [29,30]. This process forms monoiodotyrosine and diiodotyrosine that are combined to form thyroxine (T4) and triiodothyronine (T3); these THs are stored in the thyroid colloid until thyroid-stimulating hormone (TSH) induces their release into systemic circulation [29,30]. Although T3 and T4 are both secreted, the majority of T3 (≅85%) is made by deiodinase of T4 by type 1 deiodinase within hepatocytes and renal proximal tubular cells [29,31]. The hypothalamus–pituitary–thyroid (HPT) axis senses TH levels and adjusts TSH secretion and NIS expression to maintain circulating T3 and T4 within a narrow physiological range [29]. Both insufficient and excess iodine intake can eventually push these adaptive changes to a tipping point where they are no longer sufficient, causing TH production to become dysregulated [1,32].

T4 and bioactive T3 are carried throughout the body by specialized hepatic carrier proteins such as thyroxine-binding globulin, transthyretin, and albumin [29]. Thyroxine-binding globulin has the highest affinity for T4 and T3, and provides a stable binding site for these THs, whereas transthyretin is produced by the liver and regulates TH transfer across the blood–brain and placental barriers [29]. Albumin has a lower affinity for T4 and T3, but ensures efficient hormone exchange in NIS-expressing tissues such as the brain, skeletal muscle and reproductive organs, and leukocytes [31]. Only small amounts of T3 and T4 remain unbound, and therefore bioactive to interact with nuclear TH receptors and then influence gene expression [33].

Extrathymic iodine is also found in the salivary glands, gastric mucosa, small and large intestines, lactating mammary gland, kidney, ovary, prostate, and pancreas [2]. Iodide is transferred to immune-related organs such as the bone marrow, thymus, spleen, and peripheral lymph nodes that all express NISs [2]. Prolactin, estrogen, and human chorionic gonadotropin regulate placental and mammary NIS expression, ensuring that the fetus and infant receive adequate iodine during developmental stages [32]. During lactation, mammary gland epithelial cells release NISs, which transport iodide to the neonatal gastrointestinal tract and lymphoid organs, promoting offspring growth, health, and innate immune system functions [34,35]. Peripheral tissues recycle THs and remove excess iodide; the kidneys filter and eliminate 90% of iodine and iodide via urine, and less than 10% of iodine is eliminated via sweat, milk, saliva, and feces [36].

## 4. Physiological Systems Requiring Iodine

### 4.1. Iodine and Its Direct Effects on the Immune System

The mammalian immune system consists of innate and adaptive leukocytes that develop in the bone marrow or thymus and are activated in secondary lymphoid organs such as the spleen, Peyer’s patches and peripheral lymph nodes. Within these tissues, neutrophils, monocytes, macrophages, dendritic cells, T and B cells, and natural killer (NK) cells coordinate cytokine production and antigen presentation to maintain homeostasis and host microbial defences [37]. Iodine-dependent immunomodulation is likely to follow a relatively narrow optimal window, with detectable changes in leukocyte function at both deficient and excessive exposures [38]. Epidemiologic and experimental studies indicate that adequate iodine intake supports normal TH production, although the precise intake and tissue-level thresholds that define an optimal range for immune function remain uncertain [27,39,40,41].

The NIS is expressed in several extrathyroidal tissues, including the salivary glands, lactating mammary glands, gastric mucosa, thymus, reproductive organs, and immune-related organs such as the bone marrow and spleen. Its expression allows for active iodide uptake from the systemic circulation into these tissues and local exposure of leukocytes to tissue-specific iodine concentrations [1,36,38].

Human cell culture studies have shown that iodide and iodine-based compounds can directly modulate cytokine and chemokine secretion by peripheral blood mononuclear cells (PBMCs) under experimental conditions, although the relevance of these responses to in vivo immune regulation in humans needs to be clarified [27,42]. Bilal et al. 2017, found that when PBMCs were cultured with sodium iodide (NaI) at a low to sub-millimolar range (10–1000 µM), secretion of interleukin (IL)-6, interferon-γ (IFN-γ), and IL-10 increased compared with untreated cells, indicating leukocyte activation and a mixed pro- and anti-inflammatory cytokine profile [27,42,43]. However, these concentrations likely exceed typical free iodine levels within lymphoid tissues. Thus, these findings should be interpreted primarily as evidence that leukocytes can respond to elevated iodide exposure, rather than as a reflection of physiological iodide availability in vivo.

At higher iodide concentrations, or when more complex iodine formulations such as Lugol’s iodine are used, human PBMC cytokine secretion was shown to shift toward a more inflammatory profile, characterized by increased IL-8 and monocyte chemoattractant protein (MCP)-1 together with relatively reduced IFN-γ [43,44]. This suggests enhanced chemokine-driven leukocyte recruitment could occur, lowering the threshold for inflammation [43,44]. However, since these observations were derived from in vitro studies of human PBMCs under highly controlled experimental conditions, they may not fully capture whole-organism responses [35,42,43].

In vivo, iodine and iodine-mediated changes in thyroid function were also accompanied by modulated T helper (Th)1 and Th17 cell differentiation, NK cell activity, and CD8+ cytotoxic T cell responses that may influence cytotoxic and antiviral defences; however, these immunological changes were largely based on animal studies that require confirmation from well-designed human studies [44,45,46,47]. In dairy cattle, NIS and deiodinase expression were detected in mammary epithelial cells and splenocytes, supporting iodine uptake and turnover in these tissues, and potential exposure of local and recirculated leukocytes to tissue-specific iodine concentrations [25]. In support of this, a recent bovine study demonstrated that iodine supplementation was associated with reduced milk somatic cell counts, improved udder health, and enhanced Fc-γ receptor-mediated phagocytosis and antigen presentation in mammary tissues, suggesting that iodine intake in the mammary gland may affect the threshold and activation of innate inflammatory responses and adaptive immunity [25].

### 4.2. Iodine, the Endocrine System, and Indirect Immunomodulation

Leukocytes express TH receptors, TH transporters and deiodinases, indicating that they can respond directly to changes in circulating T3 and T4 [37,48]. In the context of human leukocytes, THs have been shown to modulate neutrophil chemotaxis, production of antimicrobial reactive oxygen species (ROS) and phagocytosis, and regulate major histocompatibility complex (MHC) class II expression on macrophages and dendritic cells, influence NK cell activity, and support plasma cell antibody production [49,50]. T and B cells also express TH transporters such as monocarboxylate transporter 8 and 10, and rely on T3 to upregulate glycolysis and mitochondrial respiration during cell activation, which supports their clonal expansion and differentiation into effector cells [48].

**Figure 2:**
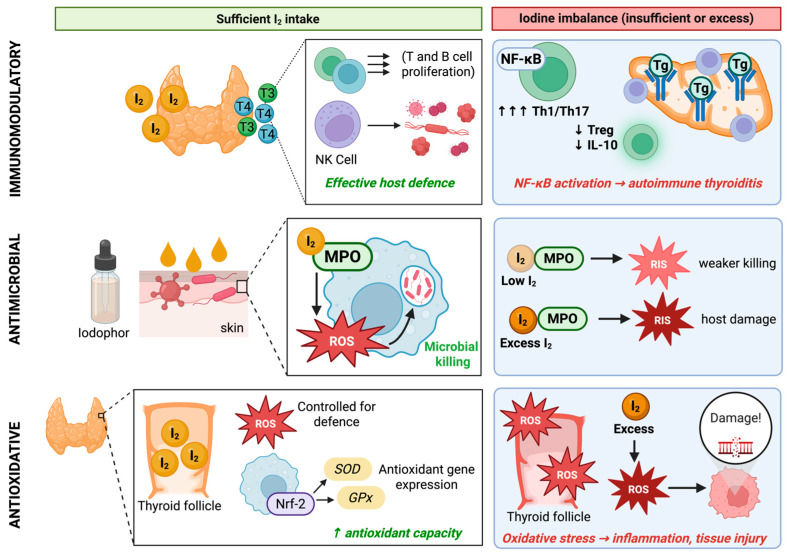
Iodine exerts immunomodulatory, antimicrobial, and antioxidative effects that depend on iodine intake and tissue context. Under sufficient iodine intake, thyroid hormone (TH) production supports balanced natural killer (NK) cell and T and B cell responses, iodophor (povidone-iodine, PVP-I) use promotes effective myeloperoxidase (MPO)-mediated microbial killing via controlled reactive iodine/oxygen species (RIS/ROS), and dietary iodide enhances nuclear factor erythroid 2-related factor 2 (Nrf2)-dependent antioxidant capacity in the blood, mammary gland, and milk, including induction of superoxide dismutase (SOD) and glutathione peroxidase (GPx). In contrast, iodine imbalance (insufficient or excess) skews T helper (Th)1/Th17 and regulatory T cell (Treg) populations with nuclear factor kappa-light-chain-enhancer of activated B cells (NF-κB) activation, predisposing to autoimmune thyroiditis, impairing MPO-derived RIS/ROS-mediated host defence at low iodine, and amplifying ROS-driven oxidative stress, inflammation, and tissue injury at excess iodine.

Also, iodine-related thyroid autoimmunity is associated with a shift in Th cells toward Th1 and Th17 cell immunophenotypes, with accompanying IFN-γ and IL-17 secretion, and reduced regulatory T (Treg) cell numbers and IL-10 secretion [46]. Together, these changes contribute to chronic inflammation and a higher risk of autoimmune flare-ups (Figure 2) [46,47]. Clinical studies have also shown that during overt hyperthyroidism, higher serum T3 and T4 levels are accompanied by elevated tumor necrosis factor (TNF)-α, increased percentages of Th1 cells, and reduced percentages of Treg cells [50,51]. In hyperthyroid Graves’ disease, PBMCs have reduced IL-2 production and impaired IL-2 receptor-mediated responses, defects that appear to normalize after the restoration of euthyroidism using the antithyroid methimazole or carbimazole drugs [52]. Some studies have also reported decreased IFN-γ production in Graves’ patients, which improved following antithyroid treatment [52,53]. These studies also described abnormal T cell regulation and suppression of T cell proliferation by thyroid follicular cells, suggesting that T cell immunity in hyperthyroidism is dysregulated, rather than uniformly increased [52,53]. Although severe thyroid dysfunction can be accompanied by increased infection risk in some patients, convincing evidence that hyperthyroidism uniformly impairs vaccine responses, or systematically increases susceptibility to common bacterial and viral infections, remains limited [54,55].

At the intracellular level, T3 and T4 have been shown to activate mitogen-activated protein kinases (MAPKs), signal transducer and activator of transcription (STAT) and NF-κB pathways in leukocytes, which may lead to changes in the expression of adhesion molecules and chemokine receptors that regulate how they migrate from the blood into lymphoid organs and inflamed tissues, but these mechanistic links still require confirmation in human studies (Figure 2) [29,49]. By modulating these signalling cascades, THs may also regulate leukocyte proliferation and cytokine production that help to adjust the efficiency of the innate and adaptive immune responses [47]. While these findings highlight iodine’s role in TH production and leukocyte function, additional genetic and environmental risk factors are typically required to elicit severe thyroid dysfunction and disease.

Reproductive physiology has also highlighted how iodine-dependent thyroid function intersects with immune development and function. Clinical data suggest that abnormal iodine and TH levels can disrupt the endometrial immune system during the implantation window, impairing uterine receptivity and increasing the risk of early pregnancy loss [20,32]. During pregnancy, maternal iodine requirements increase substantially because of higher TH production, greater renal iodine loss, and transfer of iodine to the fetus, and because of this, a daily iodine intake of about 220–250 µg for pregnant women is recommended [55,56]. Adequate iodine intake during this period allows the placenta and lactating mammary gland to supply the fetus and neonate with iodine and THs via transplacental circulation, colostrum and milk, supporting cytokine production and antibody transfer to the fetus [32,55].

In sheep, intake of iodine several-fold above recommended requirements during late pregnancy was shown to increase TH concentrations in both ewes and lambs, and was associated with lower immunoglobulin (Ig)-G concentrations in lamb plasma after consumption of colostrum, indicating impaired passive IgG transfer despite adequate colostral IgG levels [57]. Interestingly, the iodine-supplemented ewes in this study had similar or higher plasma IgG concentrations compared with the controls, so the reduced IgG in lamb plasma suggests less efficient uptake and transfer across the neonatal gut rather than a deficiency of IgG in the colostrum [57]. Follow-up work with ewes receiving excess iodine has shown concurrent changes in neonatal intestinal gene expression and barrier function, supporting the idea that maternal iodine excess can alter the neonatal gut environment in ways that limit IgG absorption, although potential mechanisms, such as altered neonatal Fc receptor (FcRn) expression, or shifts in early-life gut microbiota, have yet to be explored [57,58].

### 4.3. Other Physiological Effects of Iodine

#### 4.3.1. Antimicrobial Effects of Iodine

In addition to being required for reactive iodine species (RIS) and ROS production, iodine and iodophors are also broad-spectrum fast-acting antiseptics that have microbial effects against a variety of pathogens, including Gram-positive and Gram-negative bacteria, fungi, viruses and protozoa (Figure 2) [59]. Iodine penetrates microbial envelopes by passive diffusion, where it oxidizes and iodinates cysteine and methionine residues in structural proteins, causing denaturation and enzyme deactivation [60]. Similar oxidative and membrane-disruptive effects have been associated with iodine toxicity in mammalian cells, although the specific targets and thresholds differ from microbes [61]. Given that these iodine forms can simultaneously target multiple cellular components, there is less chance of microbes developing resistance to iodine [54].

Iodophors, such as povidone-iodine, are disinfectant solutions that bind iodine to a soluble carrier [59] that continuously releases iodine over time; this allows for a continuous iodine supply that maintains antimicrobial activity, while reducing local cytotoxicity and infections with minimal risk of cytotoxicity [52,53]. Clinical and in vitro studies have shown that iodophors retain broad-spectrum antimicrobial activity even with repeated use, and there is currently no convincing evidence that they select for bacterial resistance under clinical conditions, in contrast to many antibiotic-based agents [59]. Iodophor compositions differ according to their purpose. Applications include surgical hand washes, preoperative skin preparations and ointments. Iodophors are also used in livestock production; for example, as teat dips for dairy cattle and wound dressings to prevent infections without causing tissue damage [52,62].

#### 4.3.2. Pro- and Antioxidative Effects of Iodine

Iodine plays a key role in redox homeostasis through mechanisms that depend on its chemical form, concentration, and target tissue (Figure 2) [38,63]. In its reduced form, iodide can act as both a source and buffer of antioxidants. Iodide can directly quench ROS and, through redox-sensitive pathways such as nuclear factor erythroid 2-related factor 2 (Nrf2), promote antioxidant gene expression that limits oxidative tissue injury and alarmin release [53,64,65,66]. By enhancing these antioxidant pathways, iodide helps preserve leukocyte function in metabolically active tissues such as the thyroid and mammary gland [53,64,65,66]. Experimental studies have shown that the activation of Nrf2 by iodide can upregulate genes coding antioxidant enzymes including superoxide dismutase, catalase, and glutathione peroxidase, which may help cells neutralize ROS; however, the extent of involvement of these Nrf2-dependent pathways in humans remains to be determined in clinical studies [66,67]. In humans, both deficient and excessive iodine intake are associated with increased oxidative stress and impaired antioxidant capacity in the thyroid, whereas adequate intake supports thyroid and immune functions and limits oxidative damage [38]. In livestock, iodine supplementation at nutritionally appropriate levels supports antioxidant activity and reduces oxidative stress. In dairy cattle, for example, iodine-supplemented diets enhanced antioxidant and immune-related gene expression in the blood and mammary tissue, and reduced lipid peroxidation in tissues, milk, and cheese [25]. Experimental studies suggest that when iodine intake is excessive, iodide and its oxidized intermediates can shift the balance toward oxidative stress and cytotoxicity in susceptible tissues [38].

Enzymes such as myeloperoxidase (MPO) use hydrogen peroxide, together with iodide and other halide substrates, to generate ROS and RIS that oxidize microbial membranes and proteins (Figure 2) [54,65,67]. Thus, during iodine deficiency, reduced iodide availability limits the formation of RIS, and low TH signalling further dampens MPO expression and activation so that phagocytic cells are less effective at clearing and destroying pathogens [65,67]. Experimental models of MPO deficiency or inhibition have shown that when this metabolism pathway is compromised, pathogens are more likely to survive within phagosomes, underscoring the importance of MPO-dependent secondary oxidants for efficient microbial killing [25,65]. In companion animals, data directly linking iodine deficiency to innate immune defects are limited; however, clinical hypothyroidism in dogs is frequently accompanied by recurrent skin and external ear infections, consistent with impaired barrier immunity and altered host defence due to reduced TH production [68,69].

## 5. Immunological Outcomes of Iodine Deficiency

Iodine deficiency is associated with alterations in innate immunity. Experimental and animal data suggest that deficiency weakens myeloid cell antimicrobial responses and inflammatory signals that are needed to recruit these effector cells to infection sites [27]. Reduced TH levels in hypothyroidism are also associated with impaired neutrophil and macrophage chemotaxis, slower transendothelial migration, and attenuated production of pro-inflammatory cytokines such as IL-1, IL-6 and TNF-α [48,70,71,72].

Adaptive immune responses are also highly sensitive to iodine status because THs influence key stages of T and B cell activation, differentiation, and memory cell formation [42]. In iodine-deficient hypothyroidism, peripheral T cells show reduced proliferation in response to mitogens, diminished IL-2 and IFN-γ secretion, and lower expression of activation markers like cluster of differentiation (CD)25 and CD69 [51]. These findings support that Th1 cells and CD8+ cytotoxic T cells have impaired effector functions, with fewer cells producing IFN-γ, or upregulating activation markers, and some experimental models also show reductions in the percentages of CD4+ and CD8+ T cells [51,73,74]. These impairments are corroborated by studies reporting reduced antiviral T cell responses and slower clearance of viral infections [51,74]. Most B cell-mediated responses depend on Th cells and on TH signalling within B cells [47]. Cross-sectional studies in overt hypothyroid patients have reported higher circulating IL-6 and TNF-α compared with control patients, which appear to be normalized after levothyroxine (synthetic T4) treatment [71]. In hypothyroid patients, germinal centres within secondary lymphoid tissues formed after vaccination are typically smaller and fewer in number, and antigen-specific immunoglobulin (Ig)-G responses show several characteristic defects, including lower peak titres and delayed or incomplete class switching from IgM to IgG [54,71]. These changes reflect both suboptimal Th cell support and intrinsic limitations in B cell proliferation or differentiation, somatic hypermutation, and survival in a low-T3 environment [48,70,71,72].

## 6. Immunological Outcomes of Iodine Excess

### 6.1. Excessive Iodine and the Immune System

Excess iodine intake damages the thyroid through excessive iodination reactions that produce ROS and RIS [38,66,75]. Iodine excess, alone, does not explain all cases of thyroid dysfunction, but it is a major factor that can disrupt TH production and may lead to immune dysregulation (Table 2) [20]. Experimental and animal studies have shown that increased ROS and RIS can cause cell injury or death, and the release of alarmins that are detected by pattern recognition receptors (PRRs) expressed on macrophages, dendric cells, and epithelial cells [76]. Ligation of PRRs to alarmins leads to cell activation that initiates the NF-κB signalling cascade and transcription of several pro-inflammatory cytokines including IL-6, TNFα, and IL-β, and recruitment of inflammatory leukocytes to the thyroid [44].

Similar dose-dependent patterns during iodine excess have also been seen in domesticated animals. For example, iodine intake exceeding recommended or regulatory limits has been demonstrated to enlarge the ovine thyroid, alter TH concentrations, and negatively affect neonatal lamb growth [58]. Field and experimental reports involving sheep and cattle have also shown that such iodine excess usually presents as goitre, altered serum T3 and T4 levels, and reduced weight gain or compromised neonatal health leading to death [6,12,58]. For canids, feeding puppies commercial diets with iodine concentrations several-fold above requirements significantly reduced thyroid uptake of a diagnostic radioiodine tracer, lowered serum total and free T4 levels, increased TSH, and produced histological changes consistent with goitre; these findings show that iodine excess can potentially induce primary hypothyroidism and structural thyroid disease in dogs [68,69,79]. In cats, observational and experimental data indicate that chronically low, high, or highly variable dietary iodine intakes can alter serum TH and TSH concentrations [80,81,82]. In particular, long-term iodine excess intake has been highlighted as a potential contributor to the development of nodular thyroid hyperplasia and hyperthyroidism in older cats [80,81,82]. Examples of dietary goitrogens relevant to cats include soy isoflavones and thiocyanate-producing brassica ingredients that can interfere with TH production, especially when combined with excess iodine intake [80,81]. Environmental thyroid disruptors involved in feline hyperthyroidism include compounds such as polybrominated diphenyl ethers (PBEs), polychlorinated biphenyls (PCBs), and bisphenol A, which can be detected in household dust and are present in some canned diets [83,84].

### 6.2. Implications of the Wolff–Chaikoff Effect Versus Jod-Basedow Phenomenon on Thyroid and Immune Function

#### 6.2.1. The Systemic Influence of the Wolff–Chaikoff Effect

In thyroid tissues, a sudden increase in iodine triggers the “Wolff–Chaikoff effect”, an intrathyroidal form of autoregulation where high iodide inhibits TH production [53,63,85,86]. When intrathyroidal iodide rises above a critical threshold, excess iodide transiently inhibits thyroid peroxidase-mediated oxidation and organification of iodide, so coupling into T3 and T4 falls and new TH synthesis is drastically reduced despite abundant iodine [53,63,86]. Functionally, this means that iodine is still transported into the follicular cell and organified within the colloid, but its subsequent coupling into T3 and T4 is reduced, so new TH production is minimized even though iodine availability is high; this is known as the “blocked phase” [53,85]. In terms of the whole organism, this blocked phase acts as a short-term brake that prevents a sudden rapid increase in THs when an individual is suddenly exposed to very high iodine intake from sources such as iodinated contrast media, amiodarone, or high-dose supplements; this protects peripheral tissues from thyrotoxicosis driven typically associated with excess iodine [1,54].

The blocked phase is short-lived in a structurally and functionally normal thyroid [85,86]. The thyroid gland is not designed to remain in a blocked state for long, so it “escapes” from the inhibitory effect of excess iodide [54,86]. Mechanistically, this escape is mediated by downregulation of thyrocyte NISs. As NIS expression and activity decrease, the rate of iodide incorporation into thyroid follicular cells decreases, intrathyroidal iodide drops, and local iodide levels fall below the threshold that inhibits thyroperoxidase (TPO) [77,87]; at this point, TPO-mediated oxidation can resume, even when iodide levels remain high. In most healthy individuals with an intact thyroid reserve, the reduction in TH production during the 24–50 h blocked window results in a small and temporary reduction in circulating T3 and T4 that usually remains within homeostatic levels [54,85].

This means that protection is typically short-term and that the escape is the default outcome, and a blocking phase that persists beyond 24 to 50 h implies that the escape mechanism is impaired [88]. Since the escape mechanism happens within this short window of time, the systemic consequences in a normal and healthy thyroid are typically mild [85,89,90]. Due to their relatively long half-lives, serum TH levels change over days; T3 has a half-life of approximately 6–24 h, while the half-life of T4 is about 4–7 days [89,91]. The escape is not necessarily guaranteed, or rapid; in susceptible individuals, it may be incomplete or markedly delayed, and some patients can remain hypothyroid even after iodine is withdrawn, particularly when substantial autoimmune destruction of the thyroid has already occurred [77,88,89].

The immunological implications of the blocking phase are best understood as potential consequences of short-lived, mild hypothyroxinemia, rather than as direct effects of iodide on leukocytes [48,92]. When circulating TH levels are low, like in hypothyroidism, macrophages have reduced glycolic and phagocytic activities [87,92]. Lymphocytes also depend on TH signalling for proper activation and function, with T3 upregulating activation markers and promoting cellular expansion in response to antigens and mitogens [89,93]. Because these changes are typically observed within a 24–50 h window, these effects are likely mild, and would become more relevant if hypothyroxinemia is sustained over a longer period, because of either a failure to escape or because of underlying conditions that limit hormone levels [87,92,93].

#### 6.2.2. The Jod-Basedow Phenomenon

The Jod-Basedow phenomenon describes the development of iodine-induced thyrotoxicosis when a previously iodine-deficient or structurally abnormal thyroid is suddenly exposed to sustained iodine excess [77,88]. In contrast to the Wolff–Chaikoff effect, the Jod-Basedow phenomenon reflects a failure of this autoregulatory brake in susceptible thyroids that already have had, or currently have, nodular or autoimmune thyroid disease [1,88]. Individuals who are affected include patients with underlying thyroid conditions including those with multinodular goitre, autonomously functioning thyroid nodules, latent or overt thyroid nodules, or prior thyroid surgeries [78,86,94]. For these patients, parts of the thyroid continue to respond to iodide availability with increased TH production, but do not downregulate NIS or TPO activity when iodide accumulates or reaches the approximate 10^−3^ mol/L threshold, so they do not enter or sustain the blocked phase [77,94]. The pre-existing nodular or hyperplastic regions act as functionally autonomous tissues that produce THs with a reduced dependence on TSH and an impaired autoregulatory response to iodide [77,88]. The sudden increase in iodide availability means that the production and release of T3 and T4 are sustained, overriding pituitary negative feedback, and this results in iodine-induced thyrotoxicosis [77,88]. Data on Jod-Basedow-related thyrotoxicosis are dominated by case reports and small series in high-risk individuals exposed to contrast media or high-dose supplements; while these clearly demonstrate susceptibility in structurally abnormal thyroids, they do not define precise dose thresholds or incidence at the population level. Consequently, current estimates of Jod-Basedow risk are based on highly selected patients and do not yet define dose–response relationships or population-wide incidence.

Instead of the brief reduction in TH production and escape mechanism seen in the Wolff–Chaikoff effect, increases in free T3 and T4 and suppressed TSH are observed with the Jod-Basedow phenomenon, lasting for weeks or months if the iodine source is not removed [1,87,88]. Because the autonomous thyroid tissue continues to take up iodide as long as it is available, the thyroid can remain hyper-functional, even while the pituitary has suppressed TSH [77,94]. In many patients, this thyrotoxic state resolves once the iodine exposure ends and TH levels are reduced; however, some patients may have persistent nodular autonomy, or can transition to chronic thyroid dysfunction [77,87].

## 7. Genetic and Epigenetic Factors Contributing to Iodine Deficiency and Toxicity

Throughout this section, we highlight variants that are consistently associated with various aspects of iodine deficiency and excess. Although these variants are well established and consistent across clinical trials, in vivo and in vitro studies, effect sizes are modest and vary by ethnicity and environmental iodine exposure. Current epigenetic studies are primarily cross-sectional and use relatively small samples, limiting the ability to distinguish cause from consequence and highlighting the need for longitudinal designs that can track changes alongside exposure and disease trajectory.

### 7.1. Genetic Factors That Increase Risk of Iodine Deficiency and Toxicity

**Figure 3:**
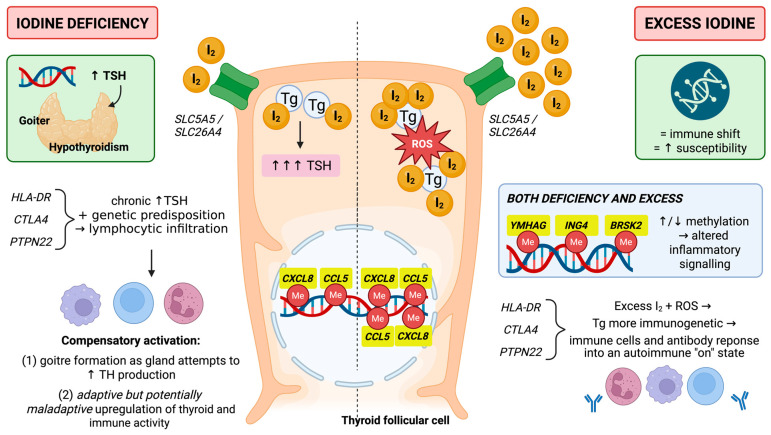
Schematic thyroid follicular cell showing how low versus high iodine (I_2_) exposure alters iodide transport (SLC5A5/SLC26A4), thyroglobulin (Tg) iodination, and oxidative stress, leading to distinct patterns of DNA methylation at chemokine genes (CCL5, CXCL8, CXCR5) and downstream immune activation. Deficiency is associated with compensatory thyroid-stimulating hormone (TSH)-driven stimulation, goitre, and hypothyroidism in genetically susceptible individuals (e.g., HLA-DR, CTLA4, PTPN22), whereas excess iodine promotes reactive oxygen species (ROS) generation, enhanced chemokine expression, and recruitment of innate and adaptive immune cells characteristic of autoimmune thyroiditis.

Globally, iodine exposure, alone, causes a subset of individuals to develop hypothyroidism, hyperthyroidism, or autoimmune thyroid disease, most commonly Graves’ disease and Hashimoto’s thyroiditis, reflecting inherited differences in iodide transport, thyroid compensation, and immune regulation that modify how thyroid and immune tissues respond to variation in iodine intake (Figure 3) [94,95,96]. These inherited differences in iodide handling influence how effectively the thyroid adapts to deficiency or excess, but these variants act alongside environmental factors rather than determining disease independently [42,85,92]. The thyroid’s ability to compensate when iodine exposure is low is dependent, in part, on soluble carrier family 5, member 5 (SLC5A5) and solute carrier family 26, member 4 (SLC26A4) [2,28,30]. SLC5A5 encodes the basolateral NIS transporter that concentrates iodide within thyroid follicular cells, whereas SLC26A4 encodes the apical pendrin anion exchanger that transfers iodide into the follicular lumen for organification onto thyroglobulin [28]. Loss of function and missense variants in these genes reduce iodide transport, limit intrathyroidal iodine accumulation, and impair TH production despite apparently adequate dietary iodine intake [95,97].

Carriers of certain SLC5A5 or SLC26A4 variants develop goitre and hypothyroidism at milder levels of deficiency, and require a higher iodine intake to avoid chronic TSH-driven overstimulation [95,97]. These transporter genes act as susceptibility modifiers rather than direct drivers of autoimmune thyroid disease, but they do not lead to loss of immune tolerance to self-antigens, a characteristic of autoimmune diseases [96,98]. Clinically, this means that individuals carrying SLC5A5 or SLC26A4 variants may reach critical thresholds for thyroid and immune dysfunction at iodine intakes that remain safe for most of the population. Since reduced TH availability can disrupt TH-dependent immune processes, variants in these genes may predispose individuals to the earlier mentioned abnormalities in lymphocyte development, migration, and cytokine production, and this warrants further investigation through human studies [96,98].

Genetic variation in immune regulatory pathways becomes more important once iodine-related thyroid stress increases the release, modification, or presentation of thyroid antigens [92,99,100]. The HLA-DR region is a primary susceptibility locus for autoimmune thyroid disease [5,33,99]. HLA-DR genes encode major histocompatibility complex (MHC) class II molecules on antigen-presenting cells, increasing the efficiency with which thyroglobulin and TPO peptides are presented to autoreactive T cells [5,33,99]. In this context, HLA-DR molecules act as upstream drivers of antigen presentation [101].

Cytotoxic T-Lymphocyte Antigen-4 (CTLA4) is another immune regulatory modifier gene that shapes the magnitude and persistence of autoreactive responses after antigen recognition has occurred [99,102]. CTLA4 encodes cytotoxic T lymphocyte-associated protein 4 (CTLA4, CD152), an inhibitory receptor expressed on activated T cells and Tregs that competes with CD28 for B7 costimulatory molecules on antigen presenting cells; thus, it acts as an “off switch” for activated T cells [102,103,104]. Functional polymorphisms such as A49G and CT60 in CTLA4 are associated with reduced CTLA4-mediated inhibition, higher thyroid autoantibody titres, and increased risk of autoimmune thyroid disease [74,90,103,104]. Thus, CTLA4 functions as a checkpoint modifier of disease progression by determining whether thyroid-reactive T cells that have already been activated are efficiently suppressed, or allowed to expand [5,99,103].

PTPN22 is another immune regulatory gene that contributes to autoreactive responses to thyroid antigens [102,105,106]. PTPN22 encodes protein tyrosine phosphatase non-receptor type 22 (PTPN22), which dampens signalling downstream of the T cell receptor [105,106]. A R620W variant of PTPN22 is associated with several autoimmune diseases, and is involved in the activation and survival of low-affinity binding self-reactive T cells [100,104,105,106]. In terms of autoimmune thyroid disease, this variant is associated with a higher risk of both Graves’ disease and Hashimoto’s thyroiditis, highlighting PTPN22 as a risk factor for susceptibility to autoreactive T and B cell responses [101,107]. Although PTPN22 is not primarily involved in antigen presentation, it acts as an immune threshold modifier that lowers the threshold under conditions of iodine-related stress [102,103].

High iodine intake interacts with these genetic risk factors to magnify autoimmune responses [40,108]. For example, excess iodine increases thyroglobulin iodination and promotes oxidative stress within thyroid follicular cells, which together enhance the generation and exposure of immunogenic thyroid peptides [21,39,64]. In individuals with autoimmune thyroid disease associated with HLA-DR alleles, heavily iodinated thyroglobulin and TPO peptides are more likely to be presented to autoreactive CD4+ T cells, so HLA-DR molecules remain the principal antigen presentation driver of disease susceptibility under iodine excess [5,97,99]. Since both CTLA4 and PTPN22 risk alleles reduce inhibitory control over these autoreactive responses, they increase the likelihood that autoreactive T cells and B cells will become activated, leading to autoantibody production and chronic infiltration of autoreactive lymphocytes into thyroid tissues [28,94,106].

### 7.2. Epigenetic Factors That Increase Risk of Iodine Deficiency and Toxicity

Epigenetic regulation, particularly DNA methylation, provides an additional layer of control over how iodine status influences thyroid and immune functions. DNA methylation refers to the covalent addition of a methyl group to cytosine residues within CpG dinucleotides in genomic DNA [109]. At promoter and enhancer regions, increased methylation is generally associated with reduced transcriptional activity, whereas hypomethylation tends to permit, or enhance, gene expression [110,111]. In immune and endocrine tissues, changes in the methylation of genes coding cytokines, chemokines, and adhesion and signalling molecules can alter leukocyte recruitment, activation thresholds, and tissue-specific stress responses without changing the DNA sequence [109,111,112]. Both iodine deficiency and excess have each been linked to characteristic methylation changes in thyroid and blood cells, indicating that iodine exposure history can leave an epigenetic imprint on immune- and apoptosis-related pathways [21,24,111].

The direct epigenetic targets of iodine status that have been implicated in autoimmune thyroiditis include the chemokine genes C-C motif chemokine ligand (CCL)-5, C-X-C motif chemokine ligand (CXCL)-8 and CXCR5, and the signalling and apoptosis-related genes Tryptophan 5-monooxygenase activation protein gamma (YWHAG), Inhibitor of Growth Family, Member 4 (ING4), Brain-Selective Kinase 2 (BRSK2), Protein Kinase AMP-Activated Catalytic Subunit Alpha 2 (PRKAA2) and Integrin Subunit Alpha 6 (ITGA6) (Figure 3) [36,111]. These loci do not function as classical germline susceptibility genes in the way that HLA-DR, CTLA4 or PTPN22 do, but instead act as iodine responsive effector and modifier genes whose methylation state translates iodine exposure history into changes in inflammatory signalling and cell survival pathways within the thyroid [111,112]. Methylation changes within these genes are better interpreted as downstream mediators that tune the intensity and persistence of autoimmune inflammation once tolerance has already been broken [24,110,111]. CCL5, CXCL8 and CXCR5 encode chemokines, which have been experimentally shown to coordinate leukocyte recruitment and positioning within inflamed tissues [111]. CXCL8 (IL-8) is a potent neutrophil chemoattractant, and CXCR5 directs B cells and T follicular helper cells into lymphoid structures [112,113]. Hypomethylation within these gene promoters shifted these genes into more transcriptionally permissive states, allowing modest inflammatory stimuli to drive stronger chemokine production and more intense lymphocyte recruitment [111]. Thus, CCL5, CXCL8 and CXCR5 do not drive autoimmunity, but rather, altered methylation provides a mechanism by which changes in iodine intake modulate thyroid lymphocyte infiltration, once autoreactive T and B cells are present [110,111].

The YWHAG, ING4 and BRSK2 genes show iodine-dependent methylation changes in patients with autoimmune thyroiditis [110,111]. YWHAG encodes the 14-3-3γ adaptor protein involved in stress and apoptosis signalling, ING4 encodes inhibitor of growth family member 4, a chromatin-associated regulator that can restrain NF-κB-driven inflammatory transcription, and BRSK2 encodes a serine kinase linked to cellular stress responses [108,110]. Across geographic regions of different levels of iodine intake, patients with autoimmune thyroiditis showed hypermethylation of YWHAG and BRSK2 and hypomethylation of ING4, with YWHAG methylation being inversely correlated with mRNA expression [73,110]. Interaction analysis further indicated that the combination of iodine fortification, YWHAG hypermethylation and BRSK2 hypermethylation increases disease risk [73,110]. In functional terms, promoter hypermethylation of YWHAG and BRSK2 is predicted to dampen the expression of stress signalling components, while hypomethylation of ING4 favours higher expression of this transcriptional regulator [110]. These methylation changes make stress and apoptosis signalling in thyroid cells less regulated, so when iodine intake is high or unstable, thyroid tissue is more likely to progress to autoimmune thyroiditis in people who are already susceptible [24].

PRKAA2 and ITGA6 are another class of iodine-sensitive epigenetic modifiers. PRKAA2 encodes the catalytic α2 subunit of adenosine monophosphate-activated protein kinase, a central regulator of energy status with downstream effects on metabolism and inflammatory signalling, while ITGA6 encodes the α6 integrin subunit [36]. In patients with autoimmune thyroiditis, both iodine supplementation after long-term deficiency and chronic iodine excess are associated with altered methylation of PRKAA2 and ITGA6 [110]. The methylation and expression changes within PRKAA2 and ITGA6 indicate that these genes respond to the iodine exposure trajectory rather than to a single intake level, acting as epigenetic markers [110]. By modifying mitogen-activated protein kinase (MAPK) signalling and integrin-mediated adhesion in response to their methylation state, PRKAA2 and ITGA6 can influence how thyroid cells and leukocytes respond metabolically and structurally to subsequent iodine status and inflammatory cues [111,114].

However, most of these epigenetic associations are derived from cross-sectional studies with relatively small sample sizes, so causality and temporal stability of the methylation patterns remain uncertain. If confirmed in larger cohorts, these iodine-sensitive patterns could eventually complement urinary iodine and circulating thyroid hormone measurements as indicators of cumulative exposure and tissue-level risk.

## 8. Testing Iodine Levels

At the population level, the urinary iodine concentration is the standard indicator [115,116]. In individuals, however, a single spot urinary iodine sample shows substantial intra-individual variability across hours and days, so one sample only approximates habitual intake [115,117]. Accuracy improves when multiple spot samples over time are combined, when urinary iodine is expressed as an iodine-to-creatinine ratio [115,116]. Direct 24 h urinary iodine excretion remains the reference method for recent iodine intake, and 24 h collections obtained during usual diet can be used to identify deficiency or recent excess exposure [115,116]. Other biomarkers can complement urinary assessment when interpreting iodine status. Serum thyroglobulin, for example, behaves as an iodine responsive biomarker in mildly deficient groups, especially during pregnancy, where it correlates with urinary iodine, and decreases after supplementation, although it is also influenced by thyroid volume and inflammation [23,118]. Standard biomarkers of thyroid function, like TSH, T4 and T3, are not direct tests of iodine status, but when interpreted alongside urinary iodine and thyroglobulin, they help distinguish low or high intake and indicate when iodine exposure has begun to alter TH production [115,118].

Because both iodine deficiency and excess are harmful, and because intake needs to stay within a relatively narrow range, objective measurement of iodine status is essential [6,10]. Population data support a U-shaped relationship between iodine intake and thyroid autoimmunity, but current reference values were designed mainly to prevent overt deficiency, and do not define a precise optimal interval for immune or epigenetic outcomes [22,23,40,92,100]. As a result, testing strategies have focused on biomarkers that describe recent iodine exposure, and the exact range for long-term thyroid and immune health remains incompletely defined [23,86,92], and warrants further research to protect both human and animal populations.

In dogs and cats, direct urinary iodine measurement is less common, and assessment often relies on diet analysis. Surveys of preprepared raw dog foods labelled as complete show that many products exceed the maximum recommended iodine levels, highlighting the potential for excess intake without specific iodine testing [68]. Under these conditions, tests of thyroid function interpreted alongside a detailed diet history are the main practical tools for recognising iodine-related thyroid risk [119]. In cats, studies of commercial foods and urinary iodine have demonstrated wide variation in dietary iodine supply and suggest that some animals experience chronically low or fluctuating intake [80,81].

## 9. Research Gaps and Future Directions

Although iodine–thyroid physiology is generally well characterized, several immunological outcomes related to iodine status remain poorly defined across humans and domesticated mammals. Most studies rely on changes in thyroid function, goitre prevalence, or growth as primary endpoints, with leukocyte function, infection burden, vaccine responses, and autoimmune activity assessed only in a subset of cohorts or in experimental models [54,120]. Many in vitro studies use artificially prepared iodide or iodine-containing solutions at low to sub-millimolar concentrations, which are likely higher and more sustained than typical free iodide levels in most tissues in vivo, and precise physiological iodide concentrations remain largely unknown [36,61]. This makes it difficult to determine the intake and tissue-level thresholds that support optimal antimicrobial defence and immune responsiveness versus those that promote chronic inflammation, oxidative stress, or autoimmune flare-ups [5,42,92].

Despite the existence of national and international iodine guidelines, recommended intakes, and the way age groups and special populations are defined, differ amongst documents from the IOM, Health Canada, and the WHO [6,7,9,18]. Even when numerical intake values are similar, the underlying life stage categories and cut-offs are not aligned [6,7,9,18]. In addition, organizations such as the WHO and the ATA provide detailed publications and policy statements on salt iodization and on the criteria used to derive iodine intake recommendations, which are valuable resources for clinicians, nutritionists, and public health practitioners [6,7,9,18]. However, many of the core intake guidelines from Health Canada, the IOM, WHO, and ATA were developed in the 1990s and early 2000s, and focus primarily on preventing classical thyroid-related iodine deficiency disorders [6,7,9,18]; they rarely incorporate explicit considerations of immune function or immune-related outcomes, highlighting the need for updated guidance that reflects evidence on iodine and immune health. Future guideline development should, therefore, explicitly integrate immune assessment endpoints into the criteria used to define safe and optimal iodine intake ranges in both humans and domesticated animals. In humans, for example, this could include longitudinal cohorts and intervention studies that quantify changes in antimicrobial activity, T and B cell numbers and responses, and vaccine outcomes across defined iodine exposure ranges [114,118]. A priority will be to identify intake ranges of iodide and molecular iodine levels that optimize immune function without increasing the incidence or severity of autoimmune thyroid disease, particularly in individuals with pre-existing thyroid conditions. In domesticated mammals, controlled supplementation and challenge experiments are also needed to test how iodine concentrations in feed or supplements influence neonatal passive transfer, mucosal barrier function and immunity, and susceptibility to infectious or autoimmune diseases in livestock and companion animals [17,68,82]. A key next step could include adjusting iodine intake within existing regulatory limits to see if this measurably improves vaccine efficacy, udder and skin health, and infection burden in livestock and companion animals. Across species, better approaches to integrating iodine intake, tissue bioavailability, and immune response endpoints will be essential for refining intake recommendations that explicitly consider immunological outcomes, rather than relying solely on the classic criteria based on iodine levels alone.

## 10. Conclusions

Optimal physiological iodine levels are within a narrow window between deficiency and excess in which thyroid, endocrine and immune functions are optimally supported. For optimal TH-dependent development, leukocyte metabolism, and MPO-mediated antimicrobial activity, sufficient amounts of iodine are required in both humans and domesticated mammals. Iodine deficiency attenuates both innate and adaptive immune functions, whereas chronic excess iodine can promote thyroid inflammation and autoimmune thyroid disease in genetically and epigenetically predisposed individuals and species. While sufficient iodine intake is necessary for normal TH production, thyroid function is not governed by iodine alone, but also by genetic susceptibility, immune-mediated mechanisms, and broader endocrine regulation. Integrating iodine intake, functional markers, and immune effects in both humans and domesticated mammals is critical for developing species- and life stage-specific recommendations that balance immune promotion with toxicity and autoimmune disease prevention. Most available data are derived from observational cohorts, case studies, and short-term experimental studies, so current recommendations rely heavily on indirect markers and require refinement. Future work should prioritise studies that combine precise iodine exposure assessment with thyroidal and immune outcomes to better define safe and effective intake ranges for vulnerable groups, including pregnant and lactating individuals, infants and young children, preterm neonates, people and companion animals with underlying thyroid autoimmunity or a history of iodine-induced thyroid dysfunction.

## Figures and Tables

**Table 1:** Comparative iodine requirements for humans and domesticated animals.

Species	Adequate Intake	Upper Limit	Reference
**Humans**	**(µg/Day)**	**(µg/Day)**	
Infants (0–6 months)	110	-	[6,7]
Infants (7–12 months)	130	-	[6,7]
Children (1–8 years)	90	300	[6,7]
Children (9–13 years)	120	600	[6,7]
Adolescents (14–18 years)	150	900	[6,7]
Adult (18–64)	150	1100	[6,7]
Pregnant women	220	1100	[6,7]
Lactating women	290	1100	[6,7]
**Companion Animals**	**(mg/kg DM)**	**(mg/kg DM)**	
Dogs	1.5	11	[10]
Cats	0.35	2.2	[10]
**Ruminants**			
Sheep—Lactating	0.50	2.5	[11,12]
Sheep—Non-lactating	0.50	10	[11,12]
Goats—Lactating	0.50	2.5	[11,12]
Goats—Non-lactating	0.50	10	[11,12]
Cattle—Beef—Adult	0.50	1.3	[13,14]
Cattle—Beef—Calf (birth–6 months)	0.50	0.7	[13,14]
Cattle—Dairy—Calf (birth–3 months)	0.25	0.7	[14,15]
Cattle—Dairy—Heifer (3 months to calving)	0.28	0.7	[14,15]
Cattle—Dairy—Dry cow (pregnant)	0.40	1.0	[14,15]
Cattle—Dairy—Lactating cow	0.50	1.3	[14,15]
Swine *	**(mg I/kg)**	**(mg I/kg)**	
Nursery pigs (5–20 kg BW)	0.14	4	[16,17]
Grower-finisher pigs (20–120 kg BW)	0.14	4	[16,17]
Gestating sows	0.14	4	[16,17]
Lactating sows	0.14	4	[16,17]
Boars	0.14	4	[16,17]

DM—dry matter; BW—body weight. * 0.14 mg I/kg (≈0.84 mg/day).

**Table 2:** Immunological consequences of excess or deficient iodine intake in humans.

Iodine Status	Immunological Consequences	
**Iodine Deficiency**	Reduced TH production. Reduced microbicidal activity and pathogen clearance. Impaired cytokine responses and leukocyte recruitment. Increased susceptibility to infections. Poor vaccine response due to weak germinal center formation. Impaired immunoglobulin class switch recombination.	[27,37,45]
**Adequate Iodine**	**Antimicrobial Effect** Supports effective phagocytosis and microbial killing. Maintains production of antimicrobial compounds. **Antioxidative and Immunomodulatory Effects** Helps maintain antioxidant enzyme activity. Contributes to balanced redox status. Limits excess inflammation.	[45,52,62]
**Excessive Iodine**	Heightened inflammatory and oxidative responses. Dysregulated T and B cell activity. Increased production of thyroid autoantibodies and risk of autoimmune disease. Disrupts TH production. **Wolff–Chaikoff Effect** Reduced chemotaxis, phagocytosis and NK cytotoxicity. Acute dampening of thyroid antigen presentation. Reduction in local inflammatory signalling. **Jod-Basedow Phenomenon** Triggers immune-mediated thyroid stimulation in predisposed glands. Increases pro-inflammatory cytokines and thyroid-reactive immune activity. Promotes development of autoimmune hyperthyroidism.	[1,41,77,78]

## Data Availability

No new data were created or analyzed in this study. Data sharing is not applicable to this article.

## Abbreviations

(BRSK2)	Brain-Selective Kinase 2
(CCL)	C-C motif chemokine ligand
(CTLA4)	Cytotoxic T-Lymphocyte-Associated protein 4
(CXC)	C-X-C motif chemokine ligand
(Th)	Helper T cell
(HPT)	Hypothalamus–pituitary–thyroid axis
(Ig)	Immunoglobulin
(ITGA6)	Integrin Subunit Alpha 6
(IFN-γ)	Interferon-gamma
(IL)	Interleukin
(ING4)	Inhibitor of Growth Family, Member 4
(MCP)	Monocyte chemoattractant protein
(MPO)	Myeloperoxidase
(MAPK)	Mitogen-activated protein kinases
(MHC)	Major histocompatibility complex
(NF-κB)	Nuclear factor-κB
(PRR)	Pattern recognition receptor
(PTPN22)	Protein tyrosine phosphatase non-receptor type 22
(PCBs)	Polychlorinated biphenyls
(PBEs)	Polybrominated diphenyl ethers
(PBMCs)	Peripheral blood mononuclear cells
(Treg)	Regulatory T cell
(PRKAA2)	Protein Kinase AMP-Activated Catalytic Subunit Alpha 2
(RIS)	Reactive iodine species
(ROS)	Reactive oxygen species
(SNP)	Single-nucleotide polymorphism
(SLC5A5)	Soluble carrier family 5, member 5
(SLC26A4)	Solute carrier family 26, member 4
(NIS)	Sodium iodide symporters
(STAT)	Signal transducer and activator of transcription
(TH)	Thyroid hormone
(TPO)	Thyroperoxidase
(T4)	Thyroxine
(YWHAG)	Tryptophan 5-Monooxygenase Activation Protein Gamma
(T3)	Triiodothyronine
(TSH)	Thyroid-stimulating hormone
(TNF-α)	Tumor necrosis factor-α

## References

[1] Khudair A., Khudair A., Niinuma S.A., Habib H., Butler A.E. (2025). Beyond Thyroid Dysfunction: The Systemic Impact of Iodine Excess. Front. Endocrinol..

[2] Gadisi R.P., Naicker M., Naidoo S. (2025). The Extra-Thyroidal Distribution of Sodium Iodide Symporter. Front. Endocrinol..

[3] Luo D., Liu Q., Wang J., Jashenko R., Ji R. (2023). Transcriptome Analysis of the Differentially Expressed Heat-Resistant Genes between *Calliptamus italicus* and *Gomphocerus sibiricus*. Environ. Entomol..

[4] Eveleigh E.R., Coneyworth L., Welham S.J.M. (2023). Systematic Review and Meta-Analysis of Iodine Nutrition in Modern Vegan and Vegetarian Diets. Br. J. Nutr..

[5] Du P., Zhu J., Yao Q., Cai T., Xu J., Fang Y., Wu Y., Zhang W., Zhang J. (2022). HLA-DRA Gene Polymorphisms Are Associated with Graves’ Disease as an Autoimmune Thyroid Disease. BioMed Res. Int..

[6] Canada H. Dietary Reference Intakes Tables: Reference Values for Elements. https://www.canada.ca/en/health-canada/services/food-nutrition/healthy-eating/dietary-reference-intakes/tables/reference-values-elements.html.

[7] Institute of Medicine (US) Panel on Micronutrients (2001). Dietary Reference Intakes for Vitamin A, Vitamin K, Arsenic, Boron, Chromium, Copper, Iodine, Iron, Manganese, Molybdenum, Nickel, Silicon, Vanadium, and Zinc.

[8] World Health Organization, Unicef (2007). International Council for Control of Iodine Deficiency Disorders. Assessment of Iodine Deficiency Disorders and Monitoring Their Elimination.

[9] Pearce E.N. (2017). The American Thyroid Association: Statement on Universal Salt Iodization. Thyroid®.

[10] Trace Minerals (2006). Nutrient Requirements of Dogs and Cats.

[11] National Research Council (US) (2007). Nutrient Requirements of Small Ruminants: Sheep, Goats, Cervids, and New World Camelids.

[12] Canadian Food Inspection Agency (CFIA) What We Heard Report—Consultation on Maximum Nutrient Values in Small Ruminant (Sheep and Goat) Feeds. http://inspection.canada.ca/en/about-cfia/transparency/consultations-and-engagement/completed/maximum-nutrient-values-sheep-and-goat-feeds/what-we-heard-report.

[13] National Research Council (2000). Nutrient Requirements of Beef Cattle: Seventh Revised Edition: Update 2000.

[14] Canadian Food Inspection Agency (CFIA) Proposal—Maximum Nutrient Values for Beef and Dairy Cattle Feeds. http://inspection.canada.ca/en/animal-health/livestock-feeds/consultations/maximum-nutrient-values-beef-and-dairy-cattle.

[15] National Research Council, Committee on Animal Nutrition, Subcommittee on Dairy Cattle Nutrition (2001). Nutrient Requirements of Dairy Cattle: Seventh Revised Edition, 2001.

[16] Canadian Food Inspection Agency (CFIA) Consultation Summary on Maximum Nutrient Values in Swine Feeds. http://inspection.canada.ca/en/animal-health/livestock-feeds/consultations/maximum-nutrient-values-swine-feeds/consultation-summary.

[17] Canadian Food Inspection Agency (CFIA) Tables of Maximum Nutrient Values for Feeds. http://inspection.canada.ca/en/animal-health/livestock-feeds/documents-incorporated-reference/tables-maximum-nutrient-values-feeds.

[18] Gerasimov G.A. (2025). Iodine Status of the Population in the WHO European Region (an Abridged Translation of Selected Sections of the WHO European Report). Probl. Endocrinol..

[19] Yamauchi I., Yabe D. (2025). Best Practices in the Management of Thyroid Dysfunction Induced by Immune Checkpoint Inhibitors. Eur. Thyroid J..

[20] Fan L., Bu Y., Chen S., Wang S., Zhang W., He Y., Sun D. (2024). Iodine Nutritional Status and Its Associations with Thyroid Function of Pregnant Women and Neonatal TSH. Front. Endocrinol..

[21] Serrano-Nascimento C., Morillo-Bernal J., Rosa-Ribeiro R., Nunes M.T., Santisteban P. (2020). Impaired Gene Expression Due to Iodine Excess in the Development and Differentiation of Endoderm and Thyroid Is Associated with Epigenetic Changes. Thyroid.

[22] Teti C., Panciroli M., Nazzari E., Pesce G., Mariotti S., Olivieri A., Bagnasco M. (2021). Iodoprophylaxis and Thyroid Autoimmunity: An Update. Immunol. Res..

[23] Iannuzzo G., Campanozzi A., Trevisani V., Rutigliano I., Abate V., Rendina D., De Filippo G. (2022). Iodine Requirements in Pediatrics: From Fetal Life to Adolescence. Front. Endocrinol..

[24] Zhou Z., Liu J., Chen Y., Ren B., Wan S., Chen Y., He Y., Wei Q., Gao H., Liu L. (2023). Genome-Wide DNA Methylation Pattern in Whole Blood of Patients with Hashimoto Thyroiditis. Front. Endocrinol..

[25] Iannaccone M., Ianni A., Elgendy R., Martino C., Giantin M., Cerretani L., Dacasto M., Martino G. (2019). Iodine Supplemented Diet Positively Affect Immune Response and Dairy Product Quality in Fresian Cow. Animals.

[26] Haggard D.L., Stowe H.D., Conner G.H., Johnson D.W. (1980). Immunologic Effects of Experimental Iodine Toxicosis in Young Cattle. Am. J. Vet. Res..

[27] Bilal M.Y., Dambaeva S., Kwak-Kim J., Gilman-Sachs A., Beaman K.D. (2017). A Role for Iodide and Thyroglobulin in Modulating the Function of Human Immune Cells. Front. Immunol..

[28] Ravera S., Reyna-Neyra A., Ferrandino G., Amzel L.M., Carrasco N. (2017). The Sodium/Iodide Symporter (NIS): Molecular Physiology and Preclinical and Clinical Applications. Annu. Rev. Physiol..

[29] Lasa M., Contreras-Jurado C. (2022). Thyroid Hormones Act as Modulators of Inflammation through Their Nuclear Receptors. Front. Endocrinol..

[30] Alotaibi H., Tuzlakoğlu-Öztürk M., Tazebay U.H. (2017). The Thyroid Na+/I− Symporter: Molecular Characterization and Genomic Regulation. Mol. Imaging Radionucl. Ther..

[31] Bruinstroop E., Van Der Spek A.H., Boelen A. (2023). Role of Hepatic Deiodinases in Thyroid Hormone Homeostasis and Liver Metabolism, Inflammation, and Fibrosis. Eur. Thyroid J..

[32] Khudair A., Khudair A., Niinuma S.A., Habib H., Butler A.E. (2025). From Deficiency to Excess: The Impact of Iodine Excess on Reproductive Health. Front. Endocrinol..

[33] Vargas-Uricoechea H., Nogueira J.P., Pinzón-Fernández M.V., Schwarzstein D. (2023). The Usefulness of Thyroid Antibodies in the Diagnostic Approach to Autoimmune Thyroid Disease. Antibodies.

[34] Moreno-Reyes R., Fuentes Peña C.F., Nuñez J.F., Sánchez M.B., Carvajal J.J., Roble K., Mendoza-León M.J., Rangel-Ramírez M.A., Opazo M.C., Lay M.K. (2025). Critical Role of Iodine and Thyroid Hormones During Pregnancy. Int. J. Mol. Sci..

[35] Wu W., Chen Y., Guo W., Zhang K., Chen W., Fu M., Pan Z., Yang Y., Zhang N., Zhang W. (2023). The Relationship Between Iodine Excess and Thyroid Function During Pregnancy and Infantile Neurodevelopment at 18–24 Months. J. Nutr..

[36] Liu J., Feng Z., Gao R., Liu P., Meng F., Fan L., Liu L., Du Y. (2024). Analysis of Risk Factors for Autoimmune Thyroid Disease Based on Blood Indicators and Urinary Iodine Concentrations. Front. Endocrinol..

[37] De Luca R., Davis P.J., Lin H.-Y., Gionfra F., Percario Z.A., Affabris E., Pedersen J.Z., Marchese C., Trivedi P., Anastasiadou E. (2021). Thyroid Hormones Interaction With Immune Response, Inflammation and Non-Thyroidal Illness Syndrome. Front. Cell Dev. Biol..

[38] Karbownik-Lewińska M., Stępniak J., Iwan P., Lewiński A. (2022). Iodine as a Potential Endocrine Disruptor—A Role of Oxidative Stress. Endocrine.

[39] Joanta A., Filip A., Clichici S., Andrei S., Cluj-Napoca Romania C.-N.R. (2006). Iodide Excess Exerts Oxidative Stress in Some Target Tissues of the Thyroid Hormones. Acta Physiol. Hung..

[40] Laurberg P., Cerqueira C., Ovesen L., Rasmussen L.B., Perrild H., Andersen S., Pedersen I.B., Carlé A. (2010). Iodine Intake as a Determinant of Thyroid Disorders in Populations. Best Pract. Res. Clin. Endocrinol. Metab..

[41] Chen X., Liu L., Yao P., Yu D., Hao L., Sun X. (2007). Effect of Excessive Iodine on Immune Function of Lymphocytes and Intervention with Selenium. J. Huazhong Univ. Sci. Technol..

[42] Cai T., Du P., Suo L., Jiang X., Qin Q., Song R., Yang X., Jiang Y., Zhang J. (2022). High Iodine Promotes Autoimmune Thyroid Disease by Activating Hexokinase 3 and Inducing Polarization of Macrophages towards M1. Front. Immunol..

[43] Wu Y., Guo Q., Liu Y., Gong X., Sun W., Li Y., Fan C., Teng W., Shan Z. (2024). Iodine Activates NLRP3 Inflammasomes in PBMCs of Patients with Autoimmune Thyroiditis and Regulates Th1 and Th17 Cell Differentiation. Endocr. Connect..

[44] Zhang Q.-Y., Ye X.-P., Zhou Z., Zhu C.-F., Li R., Fang Y., Zhang R.-J., Li L., Liu W., Wang Z. (2022). Lymphocyte Infiltration and Thyrocyte Destruction Are Driven by Stromal and Immune Cell Components in Hashimoto’s Thyroiditis. Nat. Commun..

[45] Aceves C., Mendieta I., Anguiano B., Delgado-González E. (2021). Molecular Iodine Has Extrathyroidal Effects as an Antioxidant, Differentiator, and Immunomodulator. Int. J. Mol. Sci..

[46] Yang X., Gao T., Shi R., Zhou X., Qu J., Xu J., Shan Z., Teng W. (2014). Effect of Iodine Excess on Th1, Th2, Th17, and Treg Cell Subpopulations in the Thyroid of NOD.H-2h4 Mice. Biol. Trace Elem. Res..

[47] Wenzek C., Boelen A., Westendorf A.M., Engel D.R., Moeller L.C., Führer D. (2022). The Interplay of Thyroid Hormones and the Immune System—Where We Stand and Why We Need to Know about It. Eur. J. Endocrinol..

[48] Wenzek C., Knuschke T., Hönes G.S., Boelen A., Klopfleisch R., Zwanziger D., Heuer H., Westendorf A.M., Moeller L.C., Führer D. (2025). Thyroid Hormone Receptor α Signaling Shapes Innate and Adaptive Immune Responses during Viral Infection. Eur. Thyroid J..

[49] Giuliani C., Verrocchio S., Verginelli F., Bucci I., Grassadonia A., Napolitano G. (2021). Hormonal Regulation of the MHC Class I Gene in Thyroid Cells: Role of the Promoter “Tissue-Specific” Region. Front. Endocrinol..

[50] Fang J., Yu L., Zhuang L.-G., Pei X.-Y., Wang Q., Jin G.-X. (2022). The Changes in Peripheral Blood Th17 and Treg Ratios in Hashimoto’s Thyroiditis Are Accompanied by Differential PD-1/PD-L1 Expression. Front. Endocrinol..

[51] Kishore M., Cheung K.C.P., Fu H., Bonacina F., Wang G., Coe D., Ward E.J., Colamatteo A., Jangani M., Baragetti A. (2017). Regulatory T Cell Migration Is Dependent on Glucokinase-Mediated Glycolysis. Immunity.

[52] Monstrey S.J., Govaers K., Lejuste P., Lepelletier D., Ribeiro De Oliveira P. (2023). Evaluation of the Role of Povidone-iodine in the Prevention of Surgical Site Infections. Surg. Open Sci..

[53] Uchida T., Shimamura M., Taka H., Kaga N., Miura Y., Nishida Y., Nagayama Y., Watada H. (2023). The Effect of Long-Term Inorganic Iodine on Intrathyroidal Iodothyronine Content and Gene Expression in Mice with Graves’ Hyperthyroidism. Thyroid®.

[54] Notario L., Guerrero-Espinosa E., Nistal M., Lauzurica P., Aranda A., Alemany S. (2025). Thyroid Hormone Deficiency Worsens Outcomes in Vaccinia Virus Infection. J. Virol..

[55] Censi S., Messina G., Feligiotti E., Clausi C., Piva I., Basso D., Merante Boschin I., Bertazza L., Battheu F., Barollo S. (2025). Women with Autoimmune Thyroiditis Taking Levothyroxine During Pregnancy: Is Iodine Supplementation Needed?. Nutrients.

[56] Croce L., Chiovato L., Tonacchera M., Petrosino E., Tanda M.L., Moleti M., Magri F., Olivieri A., Pearce E.N., Rotondi M. (2023). Iodine Status and Supplementation in Pregnancy: An Overview of the Evidence Provided by Meta-Analyses. Rev. Endocr. Metab. Disord..

[57] Rose M.T., Wolf B.T., Haresign W. (2007). Effect of the Level of Iodine in the Diet of Pregnant Ewes on the Concentration of Immunoglobulin G in the Plasma of Neonatal Lambs Following the Consumption of Colostrum. Br. J. Nutr..

[58] Dušová H., Trávníček J., Svoboda M., Baňoch T., Kroupová V., Peksa Z., Konečný R. (2012). The Impact of High Iodine Intake on Thyroid Function in Ewes and Lambs. Neuroendocrinol. Lett..

[59] Makhayeva D.N., Irmukhametova G.S., Khutoryanskiy V.V. (2020). Polymeric Iodophors: Preparation, Properties, and Biomedical Applications. Rev. J. Chem..

[60] Kenesheva S.T., Turganbay S., Jumagaziyeva A.B., Askhatkyzy G., Askarova D.A., Azembayev A.A., Ilin A.I., Reva O.N., Karpenyuk T.A. (2025). The Effect of Semiorganic Iodine-Containing Compounds on the Antibiotic Susceptibility of Pathogenic Microorganisms. Biomedicines.

[61] Steins A., Carroll C., Choong F.J., George A.J., He J.-S., Parsons K.M., Feng S., Man S.M., Kam C., Van Loon L.M. (2023). Cell Death and Barrier Disruption by Clinically Used Iodine Concentrations. Life Sci. Alliance.

[62] Lepelletier D., Maillard J.Y., Pozzetto B., Simon A. (2020). Povidone Iodine: Properties, Mechanisms of Action, and Role in Infection Control and *Staphylococcus aureus* Decolonization. Antimicrob. Agents Chemother..

[63] Stolc V. (1971). Stimulation of Iodoproteins and Thyroxine Formation in Human Leukocytes by Phagocytosis. Biochem. Biophys. Res. Commun..

[64] Leoni S.G., Kimura E.T., Santisteban P., De La Vieja A. (2011). Regulation of Thyroid Oxidative State by Thioredoxin Reductase Has a Crucial Role in Thyroid Responses to Iodide Excess. Mol. Endocrinol..

[65] Macvanin M.T., Gluvic Z., Zafirovic S., Gao X., Essack M., Isenovic E.R. (2023). The Protective Role of Nutritional Antioxidants against Oxidative Stress in Thyroid Disorders. Front. Endocrinol..

[66] Krishnamurthy H.K., Rajavelu I., Pereira M., Jayaraman V., Krishna K., Wang T., Bei K., Rajasekaran J.J. (2024). Inside the Genome: Understanding Genetic Influences on Oxidative Stress. Front. Genet..

[67] Céré C., Delord B., Kenfack Ymbe P., Vimbert L., Chapel J.-P., Stines-Chaumeil C. (2023). A Bacterial Myeloperoxidase with Antimicrobial Properties. BioTech.

[68] Oberbauer A.M., Belanger J.M., Petroff B.K., Brown D.E., Wolfe C.R., Famula T.R. (2025). Repeated Thyroid Function Evaluations in the Dog: A Retrospective Study of 8,309 Dogs. Front. Vet. Sci..

[69] Moravszki L., Krizsán J., Freiler-Nagy Á., Berlinger B., Elek Z., Wagenhoffer Z., Kutasi O. (2025). Assessment of Mineral Adequacy in Preprepared Raw Dog Foods Labeled as Complete. Sci. Rep..

[70] Van Der Spek A.H., Surovtseva O.V., Jim K.K., Van Oudenaren A., Brouwer M.C., Vandenbroucke-Grauls C.M.J.E., Leenen P.J.M., Van De Beek D., Hernandez A., Fliers E. (2018). Regulation of Intracellular Triiodothyronine Is Essential for Optimal Macrophage Function. Endocrinology.

[71] Lai R., Yin B., Feng Z., Deng X., Lv X., Zhong Y., Peng D. (2024). The Causal Relationship between 41 Inflammatory Cytokines and Hypothyroidism: Bidirectional Two-Sample Mendelian Randomization Study. Front. Endocrinol..

[72] Rubingh J., Spek A., Fliers E., Boelen A., Terjung R. (2020). The Role of Thyroid Hormone in the Innate and Adaptive Immune Response during Infection. Comprehensive Physiology.

[73] Limbach M., Saare M., Tserel L., Kisand K., Eglit T., Sauer S., Axelsson T., Syvänen A.-C., Metspalu A., Milani L. (2016). Epigenetic Profiling in CD4+ and CD8+ T Cells from Graves’ Disease Patients Reveals Changes in Genes Associated with T Cell Receptor Signaling. J. Autoimmun..

[74] Shi M., Liu X., Song Q., Shi Y., Tai Q. (2023). Meta-Analysis of the Rs231775 Locus Polymorphism in the CTLA-4 Gene and the Susceptibility to Graves’ Disease in Children. Open Life Sci..

[75] Kochman J., Jakubczyk K., Bargiel P., Janda-Milczarek K. (2021). The Influence of Oxidative Stress on Thyroid Diseases. Antioxidants.

[76] Li Y., Li Y., Cao X., Jin X., Jin T. (2017). Pattern Recognition Receptors in Zebrafish Provide Functional and Evolutionary Insight into Innate Immune Signaling Pathways. Cell. Mol. Immunol..

[77] Pokhrel A., Tun M.M., Miah S.S., Raina J.S., Zahedi T. (2022). A Thyrotoxicosis Surprise: Jod-Basedow Phenomenon Following IV Contrast Administration. Cureus.

[78] Campos A.D.C., Cruz Carvalho I., Sarmento S., Fonseca T. (2023). Iodine-Induced Hypothyroidism After Chemoembolization With Ethiodized Oil: A Case of Failure to Escape From Wolff-Chaikoff Effect (WCE). Cureus.

[79] Castillo V., Lalia J., Junco M., Sartorio G., Márquez A., Rodriguez M., Pisarev M. (2001). Changes in Thyroid Function in Puppies Fed a High Iodine Commercial Diet. Vet. J..

[80] Alborough R., Graham P.A., Gardner D.S. (2022). Estimating Short and Longer-Term Exposure of Domestic Cats to Dietary Iodine Fluctuation. Sci. Rep..

[81] White J.D., Malik R., Norris J.M., Malikides N. (2010). Association between Naturally Occurring Chronic Kidney Disease and Feline Immunodeficiency Virus Infection Status in Cats. J. Am. Vet. Med. Assoc..

[82] Edinboro C.H., Scott-Moncrieff J.C., Glickman L.T. (2010). Feline Hyperthyroidism: Potential Relationship with Iodine Supplement Requirements of Commercial Cat Foods. J. Feline Med. Surg..

[83] Peterson M. (2012). Hyperthyroidism in Cats: What’s Causing This Epidemic of Thyroid Disease and Can We Prevent It?. J. Feline Med. Surg..

[84] Jones B., Engdahl J.N., Weiss J. (2019). Are Persistent Organic Pollutants Important in the Etiology of Feline Hyperthyroidism? A Review. Acta Vet. Scand..

[85] Teng X., Shan Z., Chen Y., Lai Y., Yu J., Shan L., Bai X., Li Y., Li N., Li Z. (2011). More than Adequate Iodine Intake May Increase Subclinical Hypothyroidism and Autoimmune Thyroiditis: A Cross-Sectional Study Based on Two Chinese Communities with Different Iodine Intake Levels. Eur. J. Endocrinol..

[86] Bürgi H. (2010). Iodine Excess. Best Pract. Res. Clin. Endocrinol. Metab..

[87] Eng P.H.K., Cardona G.R., Fang S.-L., Previti M., Alex S., Carrasco N., Chin W.W., Braverman L.E. (1999). Escape from the Acute Wolff-Chaikoff Effect Is Associated with a Decrease in Thyroid Sodium/Iodide Symporter Messenger Ribonucleic Acid and Protein. Endocrinology.

[88] Adalja D., Kania B.E., Soliman I.M., Sanchez J., Kalatoudis H. (2023). Initially Suspected Anaphylaxis Following Iodinated Contrast: Jod-Basedow Phenomenon in a 73-Year-Old Female Without a History of Thyroid Dysfunction. Cureus.

[89] Kramer G., Hauck B.M., Chamberlain M.J. (2002). Biological Half-Life of Iodine in Adults with Intact Thyroid Function and in Athyreotic Persons. Radiat. Prot. Dosim..

[90] Tripolino O., Mirabelli M., Misiti R., Torchia A., Casella D., Dragone F., Chiefari E., Greco M., Brunetti A., Foti D.P. (2024). Circulating Autoantibodies in Adults with Hashimoto’s Thyroiditis: New Insights from a Single-Center, Cross-Sectional Study. Diagnostics.

[91] Zutinic A., Blauw G.J., Pijl H., Ballieux B.E., Westendorp R.G.J., Roelfsema F., Van Heemst D. (2020). Circulating Thyroid Hormone Profile in Response to a Triiodothyronine Challenge in Familial Longevity. J. Endocr. Soc..

[92] Kalarani I.B., Veerabathiran R. (2022). Impact of Iodine Intake on the Pathogenesis of Autoimmune Thyroid Disease in Children and Adults. Ann. Pediatr. Endocrinol. Metab..

[93] Markou K., Georgopoulos N., Kyriazopoulou V., Vagenakis A.G. (2001). Iodine-Induced Hypothyroidism. Thyroid.

[94] Dermitzaki N., Serbis A., Baltogianni M., Gialamprinou D., Giaprou L.E., Kosmeri C., Giapros V. (2025). Molecular Genetics of Primary Congenital Hypothyroidism: Established and Emerging Contributors to Thyroid Dysgenesis. Int. J. Mol. Sci..

[95] Nadeali Z., Mohammadi-Zaniani Z., Biglari S., Molavi N., Zardoui K., Mirfendereski S., Hashemipour M., Tabatabaiefar M.A., Polychronakos C. (2025). Investigating TSHR Gene Variants in Consanguineous Families: Novel Insights into Variable Expression in Familial Congenital Hypothyroidism. Front. Endocrinol..

[96] Geysels R.C., Bernal Barquero C.E., Martín M., Peyret V., Nocent M., Sobrero G., Muñoz L., Signorino M., Testa G., Castro R.B. (2022). Silent but Not Harmless: A Synonymous SLC5A5 Gene Variant Leading to Dyshormonogenic Congenital Hypothyroidism. Front. Endocrinol..

[97] Noguchi Y., Arakawa Y., Inoue N., Inaoka C., Yano Y., Hidaka Y., Iwatani Y., Watanabe M. (2026). Association of HLA-DRB1 Pocket Structures with Susceptibility and Prognosis of Graves’ and Hashimoto’s Disease. Endocr. J..

[98] Martín M., Modenutti C.P., Gil Rosas M.L., Peyret V., Geysels R.C., Bernal Barquero C.E., Sobrero G., Muñoz L., Signorino M., Testa G. (2021). A Novel *SLC5A5* Variant Reveals the Crucial Role of Kinesin Light Chain 2 in Thyroid Hormonogenesis. J. Clin. Endocrinol. Metab..

[99] Tomer Y., Huber A. (2009). The Etiology of Autoimmune Thyroid Disease: A Story of Genes and Environment. J. Autoimmun..

[100] Wang B., He W., Li Q., Jia X., Yao Q., Song R., Qin Q., Zhang J. (2019). U-Shaped Relationship between Iodine Status and Thyroid Autoimmunity Risk in Adults. Eur. J. Endocrinol..

[101] Bottazzo G., Hanafusa T., Pujol-Borrell R., Feldmann M. (1983). Role of Aberrant HLA-DR Expression and Antigen Presentation in Induction of Endocrine Autoimmunity. Lancet.

[102] Rahimova R. (2023). Relationship between CTLA4, TNF-α and PTPN22 Gene Polymorphism and the Serum Levels of Antithyroglobulin and Antiperoxidase Antibodies in Autoimmune Thyroiditis. AIMS Med. Sci..

[103] Houcken J., Degenhart C., Bender K., König J., Frommer L., Kahaly G.J. (2018). PTPN22 and CTLA-4 Polymorphisms Are Associated With Polyglandular Autoimmunity. J. Clin. Endocrinol. Metab..

[104] Thomas S.M., Veerabathiran R. (2025). Deciphering Autoimmune Susceptibility: A Meta-Analysis of PTPN22 Gene Variants. Immunol. Res..

[105] Tizaoui K., Shin J.I., Jeong G.H., Yang J.W., Park S., Kim J.H., Hwang S.Y., Park S.J., Koyanagi A., Smith L. (2022). Genetic Polymorphism of PTPN22 in Autoimmune Diseases: A Comprehensive Review. Medicina.

[106] Begovich A.B., Carlton V.E.H., Honigberg L.A., Schrodi S.J., Chokkalingam A.P., Alexander H.C., Ardlie K.G., Huang Q., Smith A.M., Spoerke J.M. (2004). A Missense Single-Nucleotide Polymorphism in a Gene Encoding a Protein Tyrosine Phosphatase (PTPN22) Is Associated with Rheumatoid Arthritis. Am. J. Hum. Genet..

[107] Wu H., Wan S., Qu M., Ren B., Liu L., Shen H. (2022). The Relationship between PTPN22 R620W Polymorphisms and the Susceptibility to Autoimmune Thyroid Diseases: An Updated Meta-Analysis. Immunol. Investig..

[108] Lafontaine N., Wilson S.G., Walsh J.P. (2023). DNA Methylation in Autoimmune Thyroid Disease. J. Clin. Endocrinol. Metab..

[109] Xiao S., Hu Y., Wang X., Yu H. (2025). Epigenetic Mechanisms in Autoimmune Thyroid Diseases: Bridging Research and Clinical Applications. Int. J. Mol. Sci..

[110] Zhou Z., Jin M., Li B., He Y., Liu L., Ren B., Li J., Li F., Liu J., Chen Y. (2023). Effects of Different Iodine Levels on the DNA Methylation of Intrinsic Apoptosis-Associated Genes and Analysis of Gene–Environment Interactions in Patients with Autoimmune Thyroiditis. Br. J. Nutr..

[111] Chen Y., Liu J., Ren B., Zhou Z., He Y., Li F., Jin M., Liu L., Wang X., Shen H. (2025). Validation of DNA Methylation and Transcriptional Characteristics in CCL5 and CXCL8 Genes in Autoimmune Thyroiditis with Varying Iodine Levels. Sci. Rep..

[112] Aldinucci D., Colombatti A. (2014). The Inflammatory Chemokine CCL5 and Cancer Progression. Mediat. Inflamm..

[113] Harrer C., Otto F., Radlberger R.F., Moser T., Pilz G., Wipfler P., Harrer A. (2022). The CXCL13/CXCR5 Immune Axis in Health and Disease—Implications for Intrathecal B Cell Activities in Neuroinflammation. Cells.

[114] Shi Y., Zhang H., Miao C. (2025). Metabolic Reprogram and T Cell Differentiation in Inflammation: Current Evidence and Future Perspectives. Cell Death Discov..

[115] Eriksson J., Barregard L., Sallsten G., Berlinger B., Weinbruch S., Manousou S., Ellingsen D.G., Nyström H.F. (2023). Urinary Iodine Excretion and Optimal Time Point for Sampling When Estimating 24-h Urinary Iodine. Br. J. Nutr..

[116] Katagiri R., Asakura K., Uechi K., Masayasu S., Sasaki S. (2016). Iodine Excretion in 24-Hour Urine Collection and Its Dietary Determinants in Healthy Japanese Adults. J. Epidemiol..

[117] Gostas D.E., Larson-Meyer D.E., Yoder H.A., Huffman A.E., Johnson E.C. (2020). Dietary Relationship with 24 h Urinary Iodine Concentrations of Young Adults in the Mountain West Region of the United States. Nutrients.

[118] Ryška A., Capdevila J., Dettmer M.S., Elisei R., Führer D., Hadoux J., Jarząb B., Locati L.D., Newbold K., Tallini G. (2025). Molecular Predictive Biomarker Testing in Advanced Thyroid Cancer—A European Consensus. Eur. Thyroid J..

[119] Allegrini G., Poirier V.J., Pezzali J.G., Beaufrère H.H., Shoveller A.K., Kopke M.A., Beeler-Marfisi J. (2024). Urinary Iodine Clearance after Iodinated Contrast Administration to Healthy Cats. J. Vet. Intern. Med..

[120] Boretti A., Banik B.K. (2022). Potential Effects of Iodine Supplementation on Inflammatory Processes and Toxin Removal Following COVID-19 Vaccination. Biol. Trace Elem. Res..

